# Supplementary material for: Native Spider Silk-Based Antimicrobial Hydrogels for Biomedical Applications
Source: Polymers (Basel). 2021 May 29;13(11):1796. doi: 10.3390/polym13111796 (PMC8198725; doi:10.3390/polym13111796)
Supplement: Supplementary file 1 [file polymers-13-01796-s001.zip › polymers-1236283-supplementary.pdf]

Supporting Information

Native Spider silk based antimicrobial hydrogels for biomedical applications

Sinith Withanage, Artemii Savin, Valeria Nikolaeva, Aleksandra Kiseleva, Marina Dukhinova, Pavel Krivoshapkin, Elena Krivoshapkina*

SCAMT Institute, ITMO University, Lomonosova str.9, Saint Petersburg, 191002, Russian Federation

Table of Contents

1.0 FTIR – Spectroscopy 3
2.0 Swelling Degree 4
3.0 Shrinking ability 5
4.0 Drug loading and drug release 6
5.0 Conductivity 8
6.0 Enzymatic degradability 9
7.0 Antimicrobial ability 11
8.0 Cytotoxicity 14

1.0 FTIR – Spectroscopy

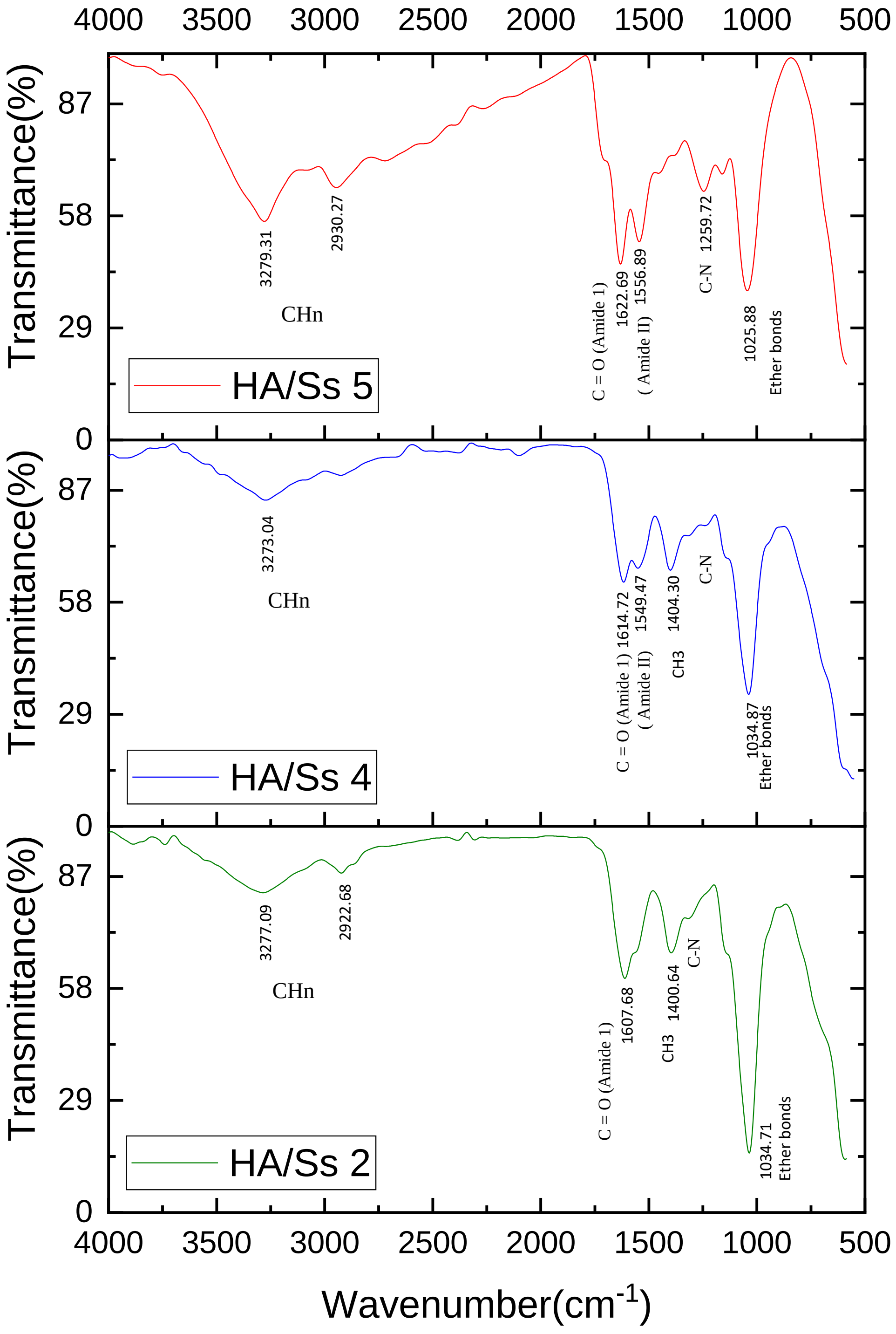

Figure S1:- FTIR – spectroscopy of HA/Ss 2, HA/Ss 4 and HA/Ss 5 hydrogel preparations

2.0 Swelling Degree

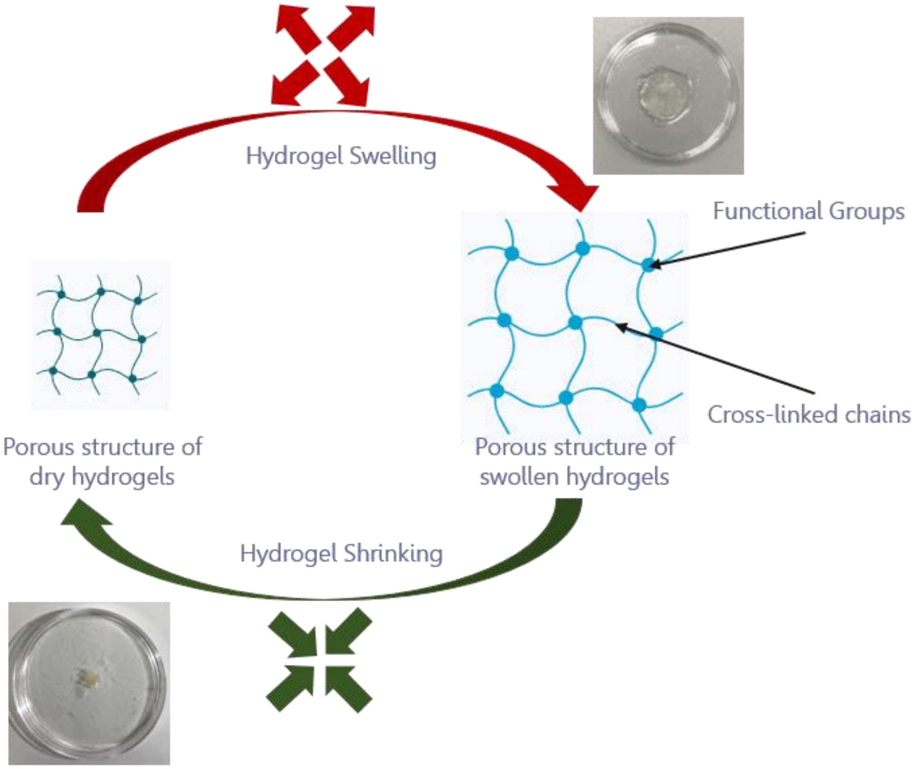

Figure S2: Schematic swelling mechanism of HA/Ss hydrogels

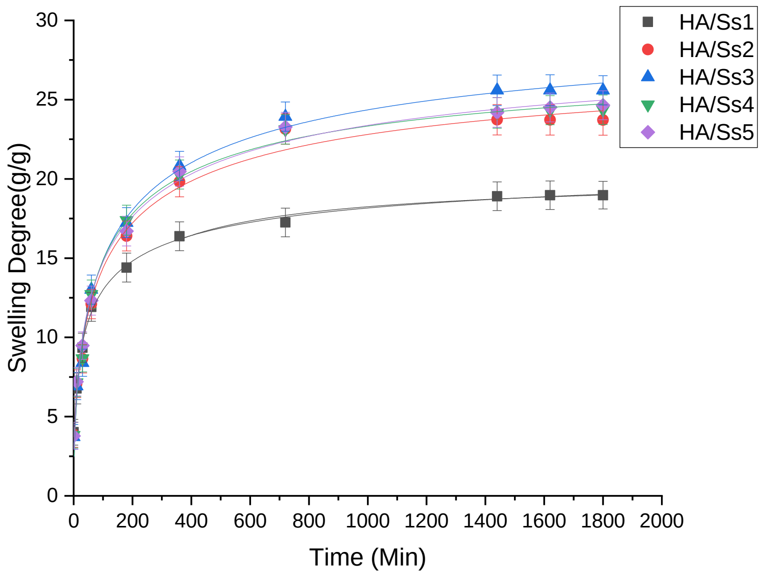

Figure S3: Swelling ability of HA/Ss hydrogels according to different component ratios

3.0 Shrinking ability

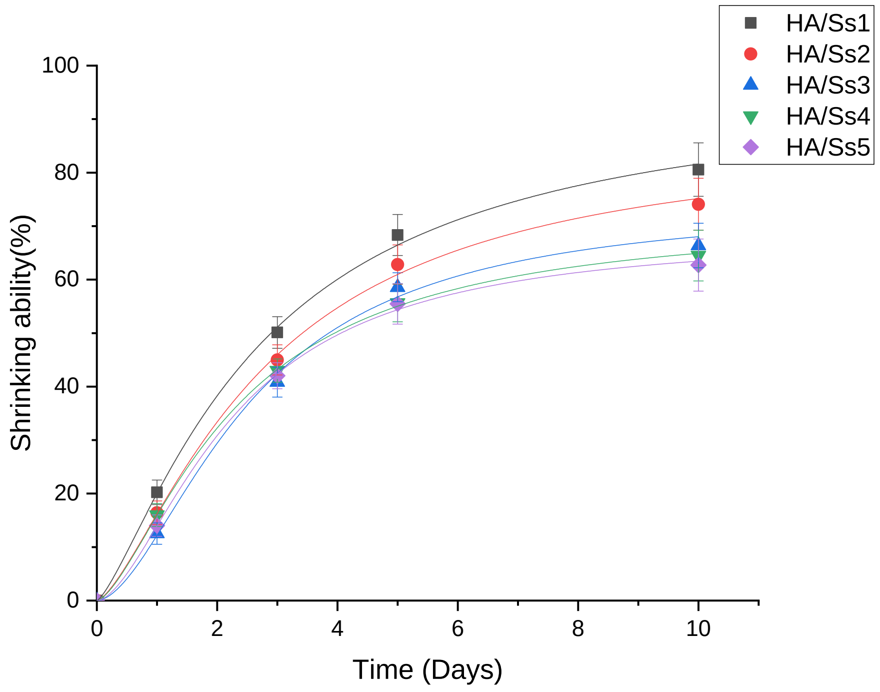

Figure S4: Shrinking ability of HA/Ss hydrogels according to different component ratios

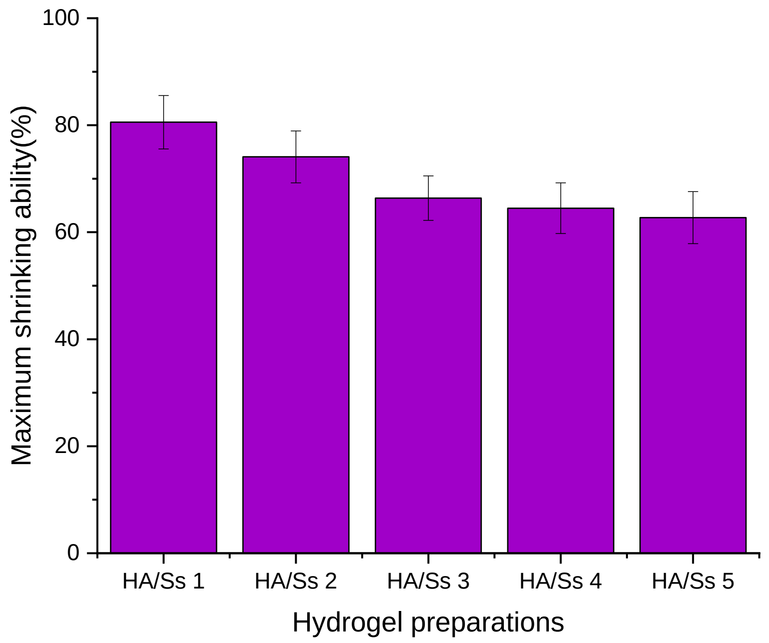

Figure S5: Maximum shrinking ability of HA/Ss hydrogels according to different component ratios

4.0 Drug loading and drug release

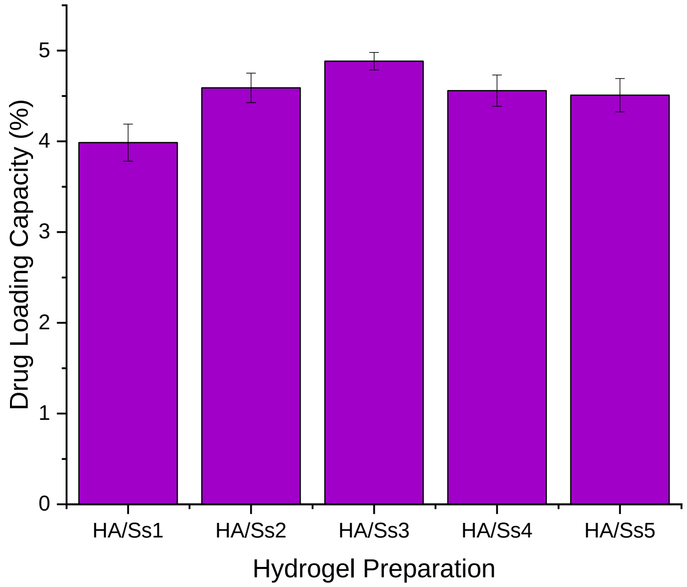

Figure S6: Drug loading capacity (%) (Ibuprofen) of HA/Ss hydrogels according to different component ratios

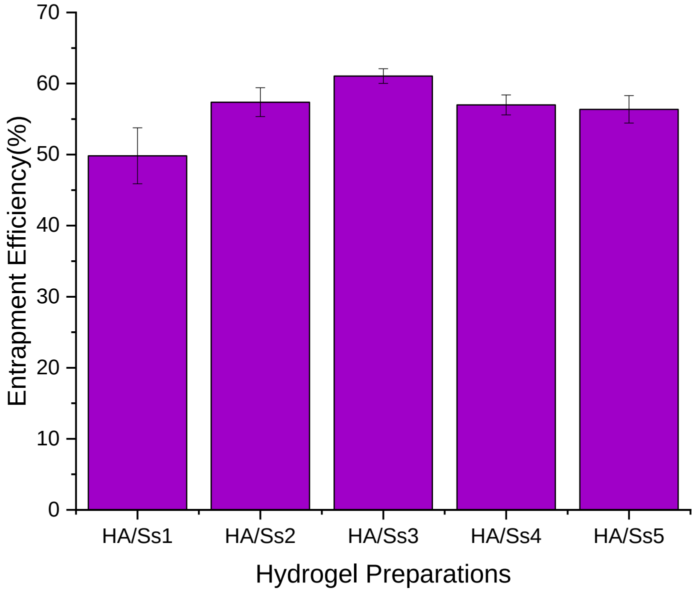

Figure S7: Drug entrapment efficiency (%) (Ibuprofen) of HA/Ss hydrogels according to different component ratios

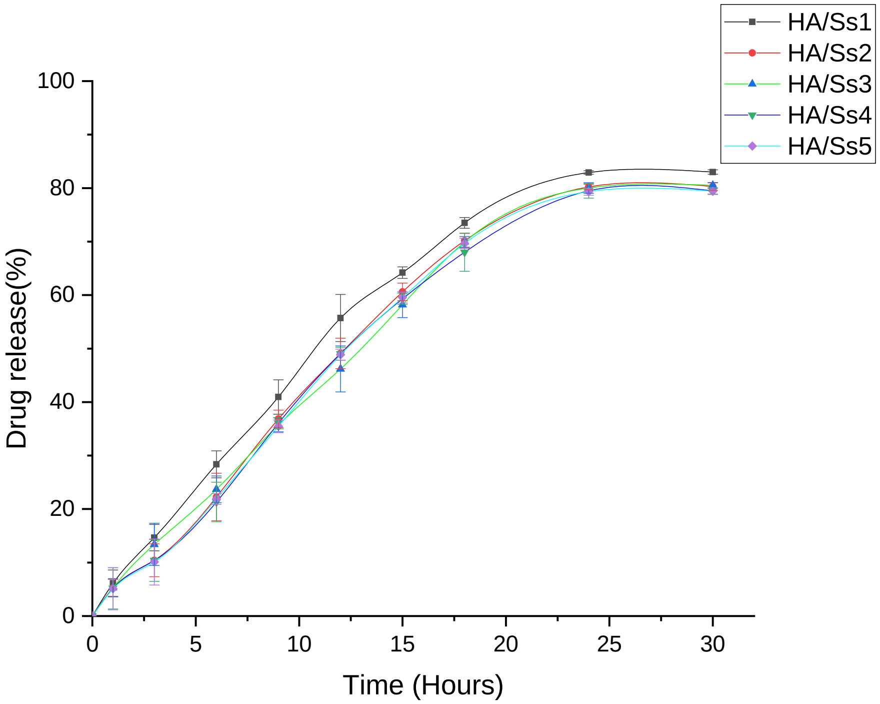

Figure S8: Drug (Ibuprofen) release of HA/Ss hydrogels according to different component ratios

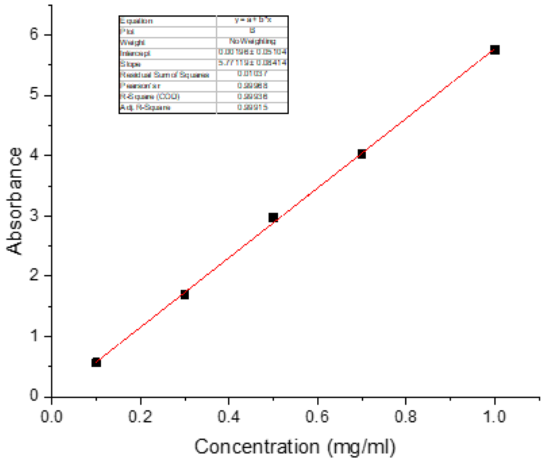

Figure S9: Ibuprofen calibration curve with UV-Vis spectroscopy under 221nm wavelength

5.0 Conductivity

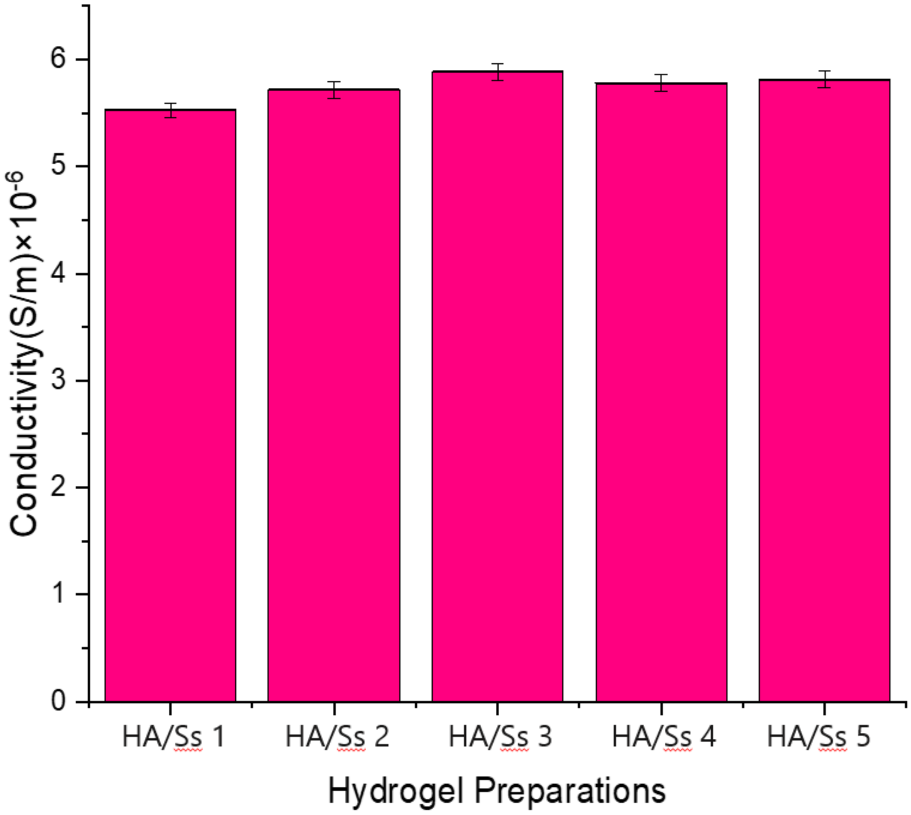

Figure S10: Conductivity of HA/Ss hydrogels according to different component ratios

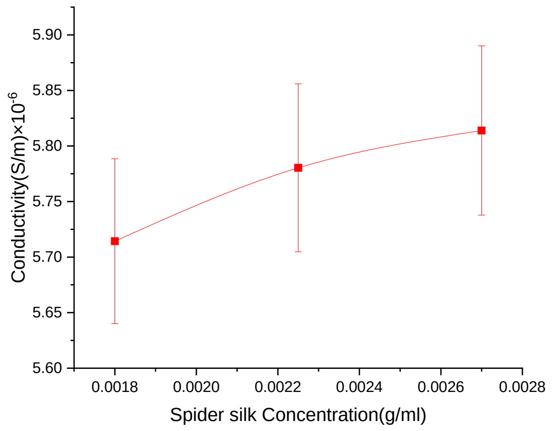

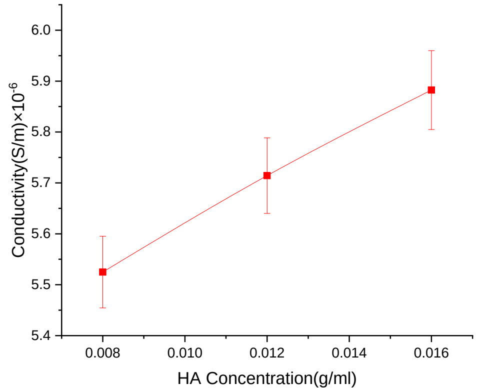

Figure S11: Conductivity of the hyaluronic acid/ Spider silk based hydrogels according to the spider silk concentration in hydrogels and hyaluronic acid concentration in hydrogels

6.0 Enzymatic degradability

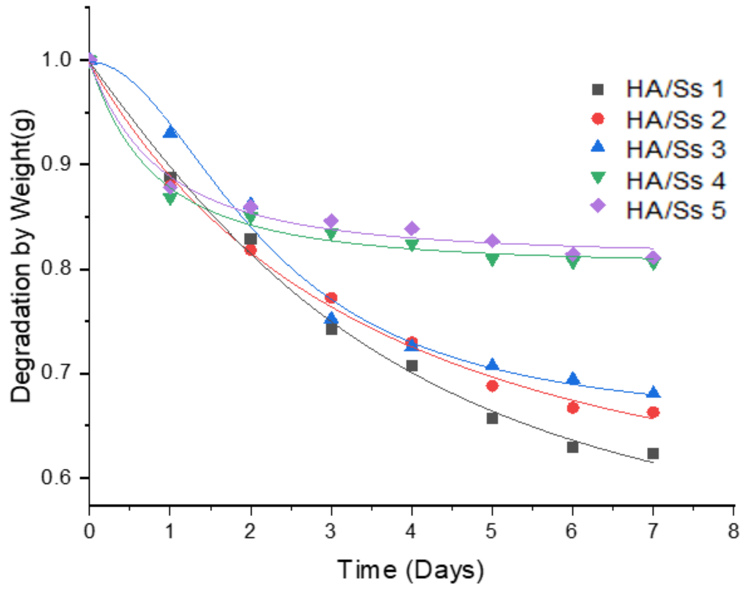

Figure S12: Degradation by weight of HA/Ss hydrogels with enzyme Chymosine over 7 days of duration (According to different component ratio preparations)

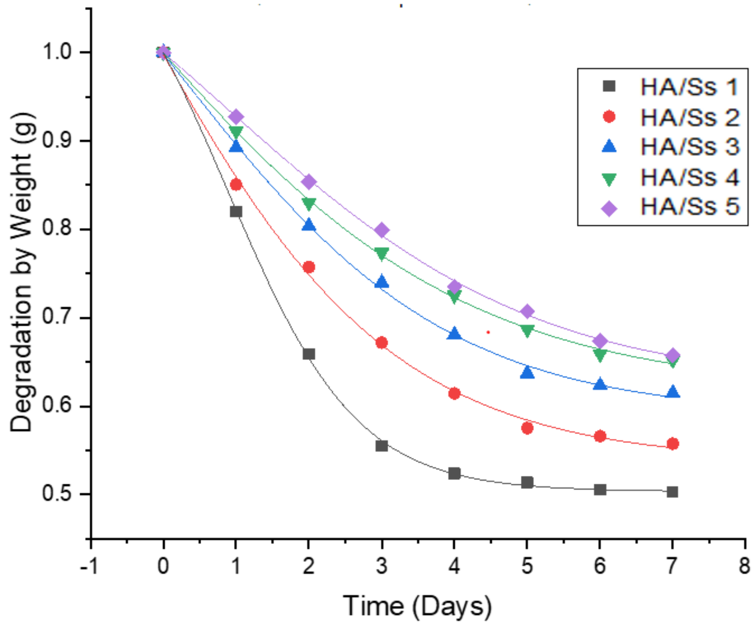

Figure S13: Degradation by weight of HA/Ss hydrogels with enzyme Trypsin over 7 days of duration (According to different component ratio preparations)

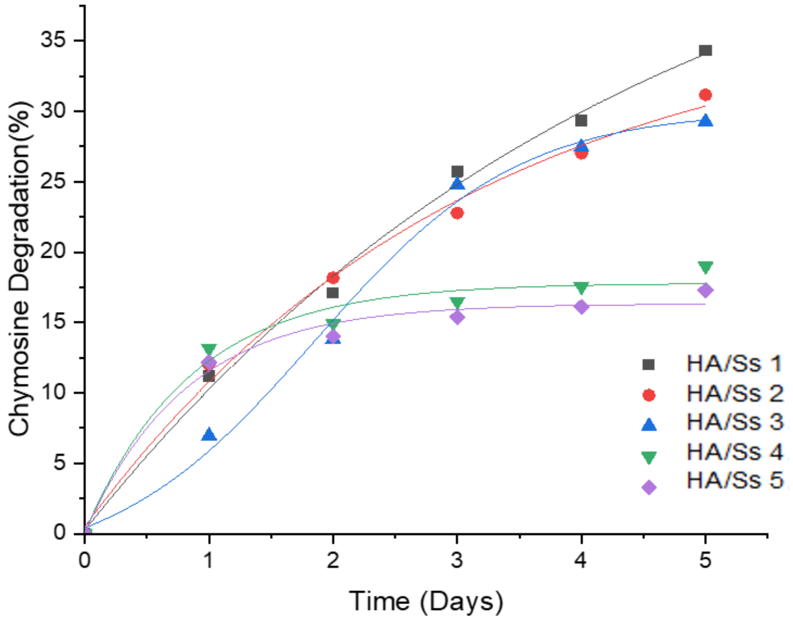

Figure S14: Degradation percentage of HA/Ss hydrogels with enzyme Chymosine compared to their initial weight (According to different component ratio preparations)

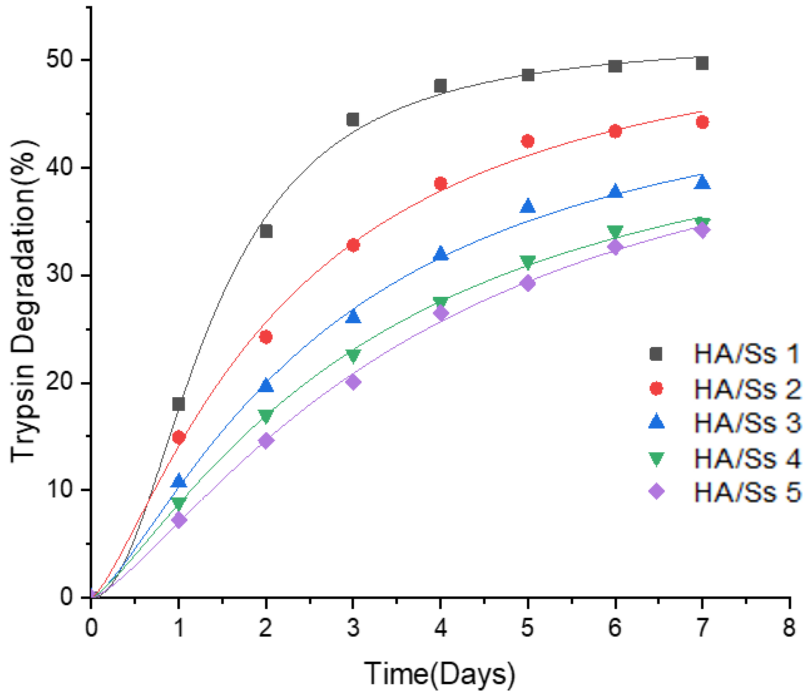

Figure S15: Degradation percentage of HA/Ss hydrogels with enzyme Trypsin compared to their initial weight (According to different component ratio preparations)

7.0 Antimicrobial ability

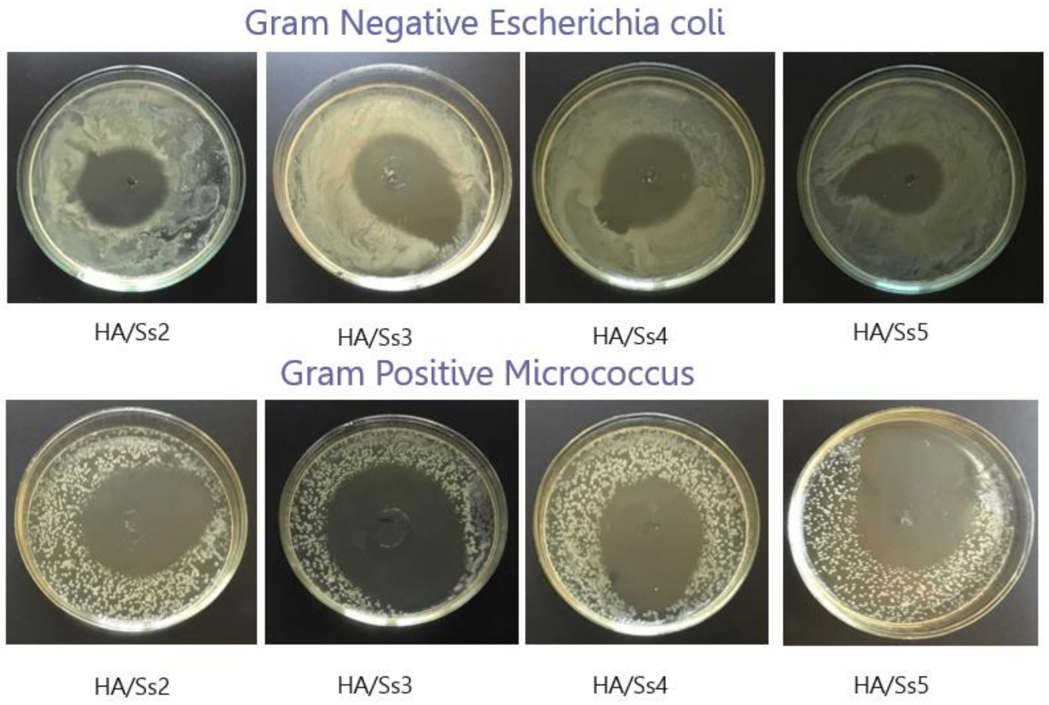

Figure S16: Antimicrobial ability of hyaluronic acid/ spider silk based hydrogels in different component ratios

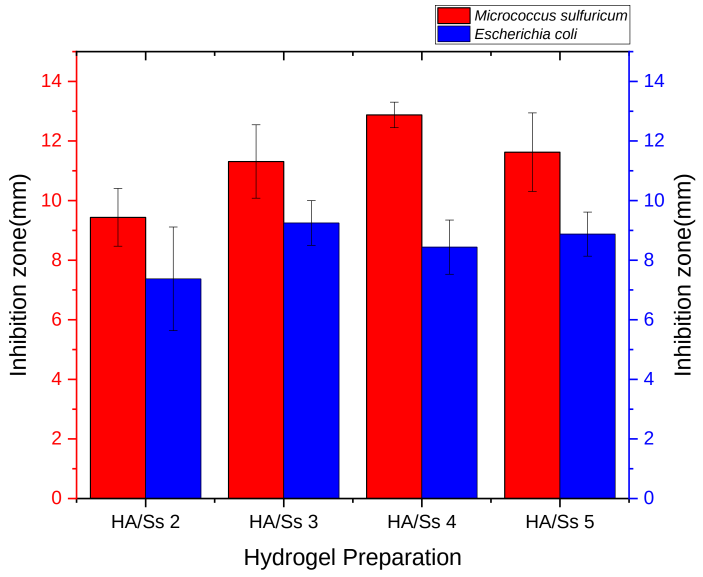

Figure S16: Antimicrobial ability of hyaluronic acid/ spider silk based hydrogels in different component ratios (Inhibitory zone parameters)

7.1 Concentration dependent effects on the antimicrobial ability (Hyaluronic acid and Spider silk)

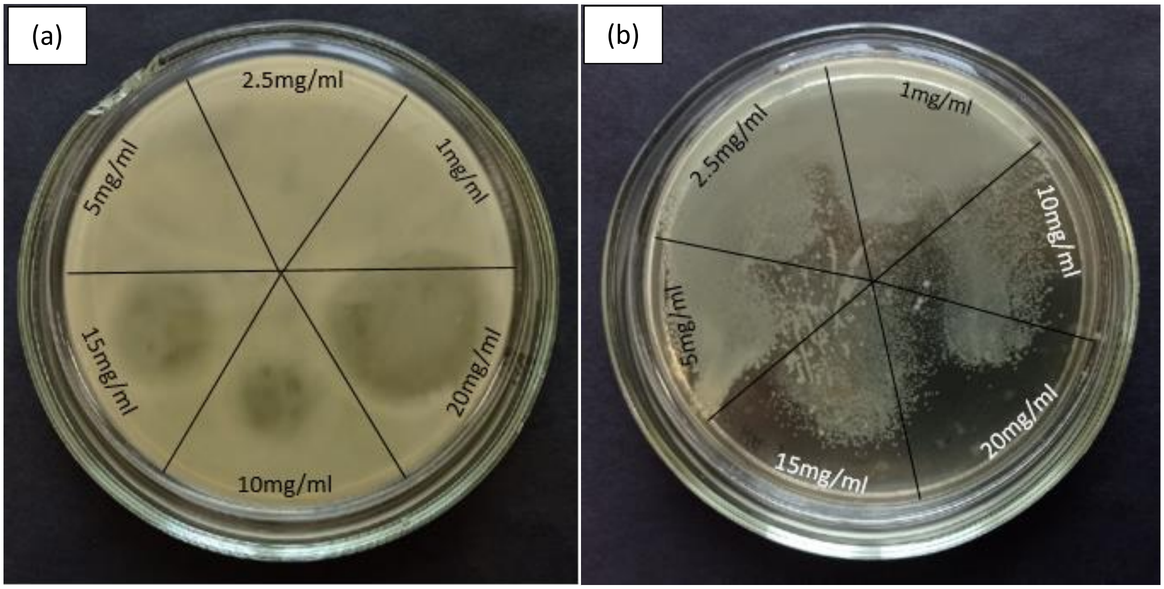

Figure S17: (a) Concentration dependent hyaluronic acid antimicrobial ability on Escherichia coli (b) Concentration dependent hyaluronic acid antimicrobial ability on Micrococcus sulfuricum

Hyaluronic acid shows concentration dependent bacteriostatic inhibitory antibacterial properties on both gram negative and gram positive bacteria.

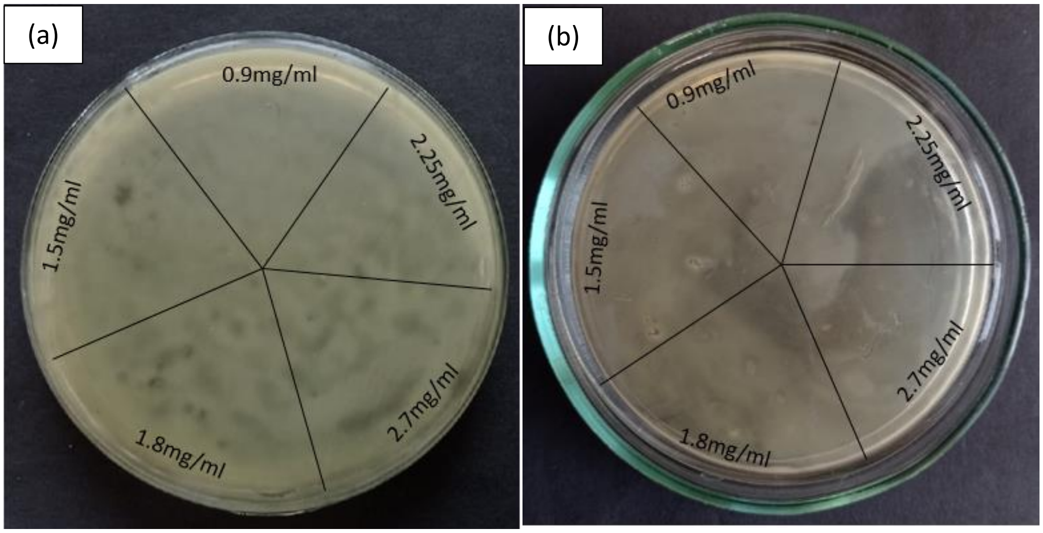

Figure S18: (a) Concentration dependent spider (silk solution) antimicrobial ability on Escherichia coli (b) Concentration dependent spider silk (solution) antimicrobial ability on Micrococcus sulfuricum

Spider silk solution in the shown concentrations does not show drastic antibacterial effects. Besides, in the range of hydrogel preparation component ratios (1.8mg/ml – 2.7mg/ml) spider silk solution shows extremely slight antibacterial effects which can assume that the silk solution has slight properties on anti-adhesion of bacteria.

7.2 Bacterial adhesion on Hyaluronic acid/ Spider silk based hydrogel surfaces

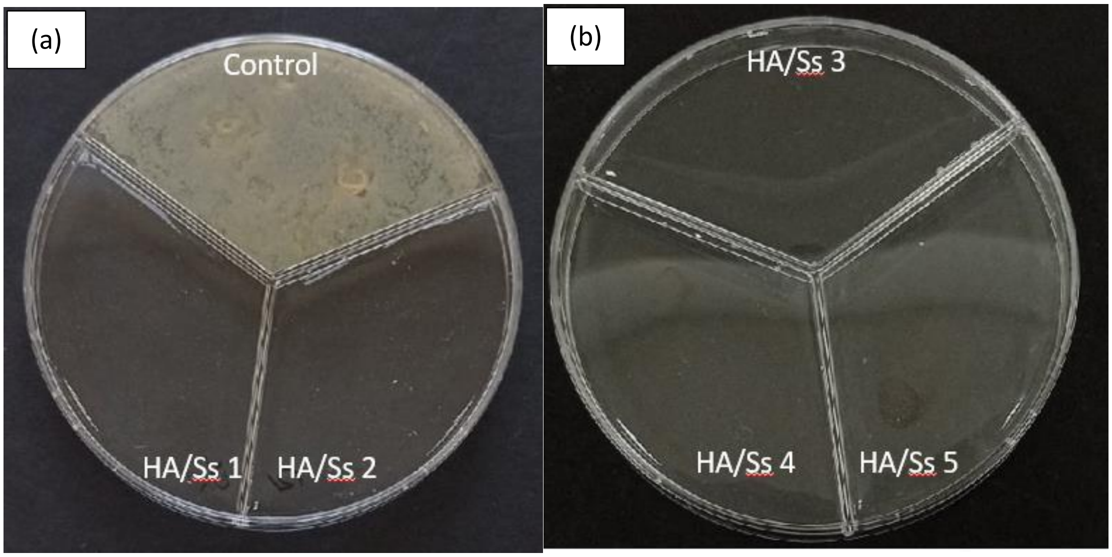

Figure S19: (a) Escherichia coli cultured on the hydrogel surfaces of HA/Ss 1, HA/Ss 2 and control (agar) mediums. (b) Escherichia coli cultured on the hydrogel surfaces of HA/Ss 3, HA/Ss 4 and HA/Ss 5.

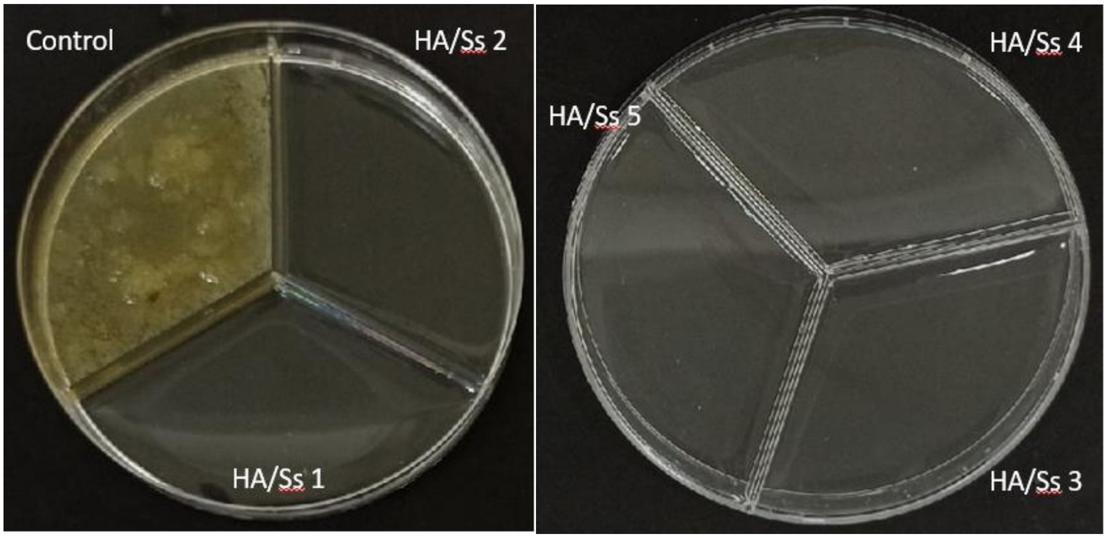

Figure S20: (a) Micrococcus sulfuricum cultured on the hydrogel surfaces of HA/Ss 1, HA/Ss 2 and control (agar) mediums. (b) Micrococcus sulfuricum cultured on the hydrogel surfaces of HA/Ss 3, HA/Ss 4 and HA/Ss 5.

Bacterial growth on the hydrogel surfaces shows lack of bacterial attachment on the hydrogels surfaces compared to the control sample with agar. There was no such profound colonies on hydrogel surfaces except some isolated colonies. The results suggest that combination of the hyaluronic acid and spider silk inhibits the bacterial attachment with the bacteriostatic and anti-adhesive properties.

8.0 Cytotoxicity

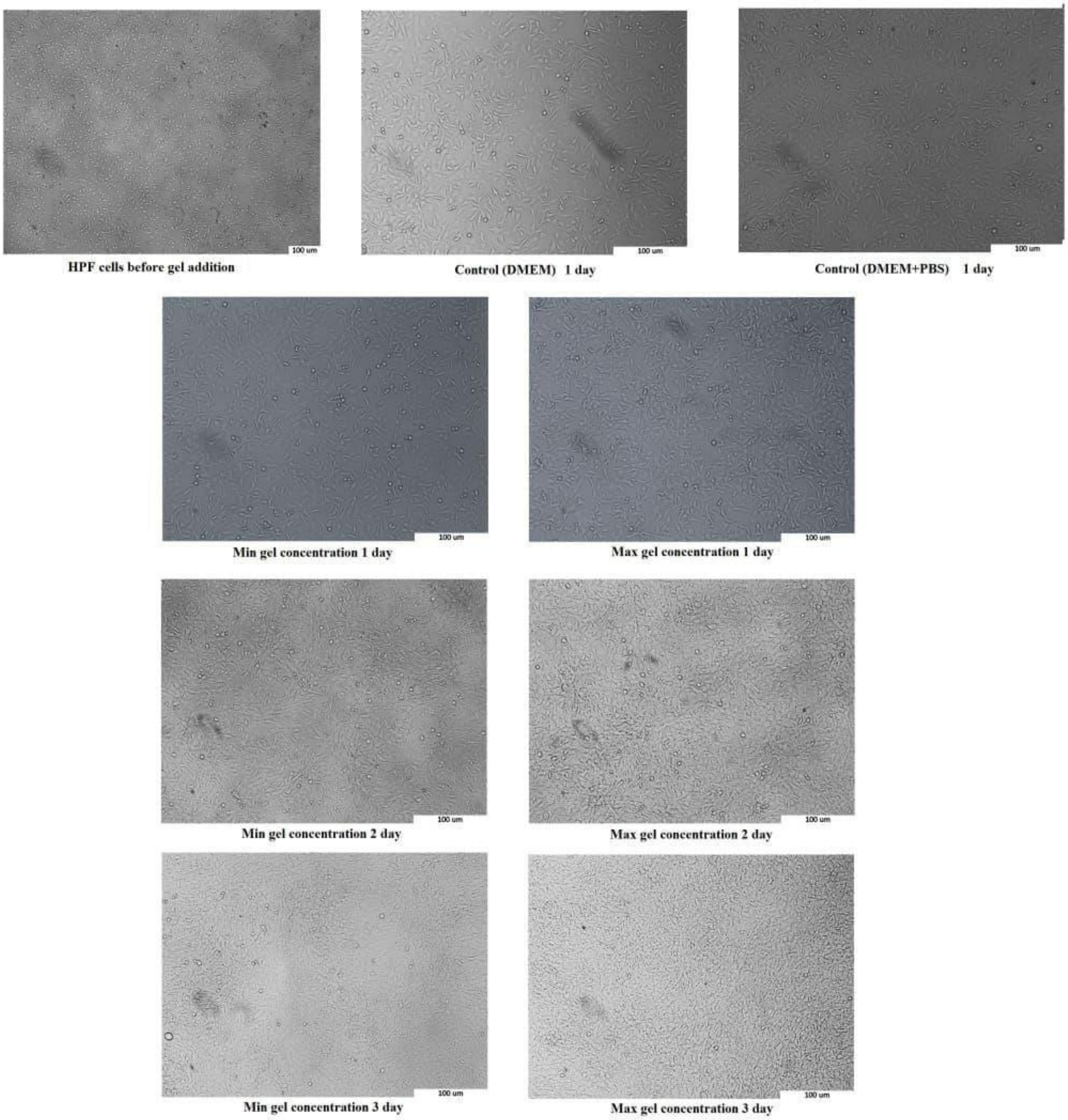

Figure S 21: Human postnatal fibroblast cell viability on High gel concentration (HA concentration = 15 mg/ml; Spider silk concentration = 3 mg/ml) and Low gel concentration (HA concentration =0.12 mg/ml; Spider silk concentration = 0.025 mg/ml) compared to the control (DMEM+PBS) and control (DMEM).

The cell morphology was not affected and observed the normal cell morphology in comparison with the control preparations.
